# Distribution of *EGFR* fusions in 35,023 Chinese patients with solid tumors-the frequency, fusion partners and clinical outcome

**DOI:** 10.1186/s12957-024-03463-w

**Published:** 2024-07-25

**Authors:** Haiping Zhang, Julei Wang, Xiaoxiao Li, Dongfeng Zhang, Yingxue Qi, Qin Zhang, Ningning Luo, Xiaoou Wang, Tuo Wang

**Affiliations:** 1 Department of Thoracic Surgery, The First Affiliated Hospital of Xinjiang Medical University, Urumqi, China; 2 Department of Neurosurgery, Second Affiliated Hospital of Air Force Military Medical University, Xi’an, China; 3 Department of Thoracic Surgery, The First Affiliated Hospital of China Medical University, Shenyang, China; 4 Department of Thoracic Oncology, Linfen Center Hospital, Linfen, China; 5 Jiangsu Simcere Diagnostics Co., Ltd, Nanjing Simcere Medical Laboratory Science Co., Ltd, The State Key Laboratory of Neurology and Oncology Drug Development, Nanjing, China; 6 Health Education Promotion Department, Shenyang Stomatological Hospital, Shenyang, China; 7 Department of Neurosurgery, The First Affiliated Hospital of Xi’an Jiao Tong University, No. 277, Yanta West Road, Xi’an City, Shanxi Province, China

**Keywords:** *EGFR* fusion, Next-generation sequencing, Solid tumors, Molecular characteristics, Clinical outcome

## Abstract

**Background:**

Epidermal growth factor receptor (*EGFR)* fusions are rare but potentially actionable oncogenic drivers across multiple solid tumors. However, the distribution and molecular characteristics of *EGFR* fusions in Chinese patients with solid malignancies have not been explored.

**Methods:**

Panel-based next-generation sequencing (NGS) data of 35,023 patients with various types of solid tumors was collected and analyzed from the Simcere Diagnostics (Nanjing, China) database. A 9563-patient cohort was derived from The Cancer Genome Atlas (TCGA) to explore the relationship between *EGFR* fusion status and overall survival (OS).

**Results:**

In this study, prevalence of functional *EGFR* fusions was 0.303% (106/35,023) in total across solid tumors, which occur more commonly in gastroesophageal junction cancer (1/61, 1.613%), followed by medulloblastoma (1/66, 1.515%) and glioma (33/2409, 1.370%). Analysis showed a prevalence for fusion partners in different tumor types. The top 3 co-mutant genes with *EGFR* fusion were *TP53* (mutation frequency, MF: 65%), *BRCA2* (MF: 43%), and *ALK* (MF: 41%). Furthermore, patients in the *EGFR* fusion group had a significantly shorter OS than those in the non-*EGFR* fusion group (*p* < 0.0001) in the TCGA cohort, suggesting that *EGFR* fusion might be a high-risk factor for poor prognosis.

**Conclusions:**

Our study is the first retrospective analysis of *EGFR* fusions in a large-scale solid tumor population, which may provide a reference for future EGFR-TKI clinical trials with *EGFR* fusions.

**Supplementary Information:**

The online version contains supplementary material available at 10.1186/s12957-024-03463-w.

## Introduction

Epidermal growth factor receptor (EGFR) is a receptor tyrosine kinase that belongs to the ERBB protein family, which includes three other members, namely ErbB2/HER-2, ErbB3/HER-3 and ErbB4/HER-4 [1–3]. Overexpression of EGFR or activation through ligand-dependent and ligand-independent mechanisms are common driving mechanisms in cancer [3, 4]. Subsequently, a cascade of multiple events in the cytoplasm occurs, leading to cell proliferation, survival, and inhibition of apoptosis [1, 5]. The key role of EGFR in cell signaling pathways makes it a major therapeutic target for cancer [6–8]. EGFR tyrosine kinase inhibitors (TKIs), as single-target inhibitors of EGFR, have completely changed the treatment model for patients with non-small cell lung cancer (NSCLC) [9, 10]. To date, many different small molecules have been developed as potential EGFR TKIs, from first to fourth generation, some of which are used in the clinical treatment of cancer [10–13].

*EGFR* mutations occur more commonly in lung cancer and glioma. In NSCLC, the common mutation of *EGFR* is located in exons 18–21. Deletions in exon 19 and single amino acid substitutions in exon 21 to L858R, commonly referred to as “classic” *EGFR* mutations, together account for approximately 80-85% of *EGFR* mutations observed in NSCLC [14–16]. In glioma, the most common cause of abnormal activation of the *EGFR* pathway is *EGFR* amplification and mutation [17]. The most common *EGFR* mutation in glioma is *EGFRvIII* mutation caused by deletion of exons 2–7. The incidence of *EGFRvIII* mutations in primary glioblastoma is only about 20–30%. Both *EGFR* amplification and the occurrence of *EGFRvIII* mutations predict a poor survival prognosis in glioma patients [18].

As detection technology advances, some rare or atypical *EGFR* mutations have been identified. Gene fusions involving *EGFR* are rare in various cancers. One study reported the discovery of 5 *EGFR* fusions in analysis of 10,000 NSCLC case [19]. Kartik Konduri et al. first reported carcinogenic *EGFR* fusion in lung cancer, the most common being *EGFR-RAD51* [20]. Subsequently, several cases of *EGFR* fusion were reported in lung cancer, colorectal cancer, glioblastoma, and other cancers, of which lung cancer was the most common. The patients with *EGFR* fusion can benefit from the corresponding EGFR-TKIs, including erlotinib, icotinib, and afatinib [21–24]. The mechanism of *EGFR* fusion in tumors remains unclear, and EGFR fusion could be a potential target.

In this study, we molecularly characterized 35,023 Chinese patients’ tumor samples across multiple solid tumors by next-generation sequencing (NGS) and further analyzed the data of 106 patients with *EGFR* fusions. The aim is to provide an outlook for patients with *EGFR* fusions in solid tumors and evidence for more effective treatment.

## Methods

### Patient information and sample collection

A total of 35,023 patients with 13 cancer types diagnosed between January 2019 and December 2022 were enrolled in this study. The cohort underwent comprehensive genomic profiling of the targeted panel by Simcere Diagnostics Co., Ltd. (Nanjing, China), of which 14,874 patients were detected using a 539-gene panel. The study was conducted in accordance with the Declaration of Helsinki. The written informed consent was waived for this retrospective analysis.

### Next-generation sequencing detection

#### DNA extraction and library preparation

Formalin-fixed paraffin-embedded (FFPE) tumor slides and paired blood samples were collected. Two DNA extraction kits were used: a Tissue Sample DNA Extraction Kit (Kai Shuo) for genomic DNA (gDNA) extraction from tumor tissue and a MagMAXTM DNA Multi-Sample Ultra Kit (Thermo) for gDNA extraction from leukocytes. The extraction procedures were performed according to the manufacturer’s protocol. Library construction used the probe hybridization capture method. Briefly, 15–200 ng gDNA was fragmented into 200 ~ 350 bp fragments by fragmentation enzymes. After end repair, poly(A)-tailing and adaptor ligation, customized probes and a commercial kit were used for hybridization capture and library quantification, respectively.

### Library sequencing and bioinformatics analysis

The qualified DNA libraries were sequenced on an Illumina NovaSeq6000 platform (Illumina, San Diego, CA) to generate 150 bp paired-end reads. Base calls from Illumina NovaSeq6000 were conducted to FASTQ files. The software Fastp (v.2.20.0) was used for adapter trimming and filtering of low-quality bases [25]. The BWA-MEM (v.0.7.17) algorithm was performed to align to the reference genome (UCSC hg19 GRCh37) [26]. Duplicate reads from PCR were excluded using Dedup with Error Correct. SNVs/InDels were called and annotated via VarDict (v.1.5.7) [27] and InterVar [28]. The variants were filtered against the common SNPs in the public database, including the 1000 Genome Project (Aug 2015) and Exome Aggregation Consortium (ExAC) Browser28 (v.0.3). Copy number variations (CNVs) and fusions were analyzed by CNVkit (dx1.1) [29] and factera (v1.4.4) [30], respectively.

TMB was defined as the number of somatic, coding, base substitution, and indel mutations per megabase of genome examined. The 539-cancer gene-targeted NGS panel TMB was counted by summing all base substitutions and indels in the coding region of targeted genes, excluding synonymous alterations, alterations of AF < 0.02, and alterations listed as known somatic alterations in COSMIC.

### Data acquisition and analysis from TCGA

All clinical and genomic data of 9563 solid tumors across 21 tumor types were retrieved from cBioPortal (www.cbioportal.org). These data were collected from The Cancer Genome Atlas (TCGA) Pan-Cancer analysis project [31]. The CNV pipeline and pipelines for the detection of gene fusions are described on the GDC documentation website (https://docs.gdc.cancer.gov/Data/Introduction/).

### Statistical analysis

All statistical analyses were performed using R V4.0.5 (https://www.r-project.org). Differences in TMB between subgroups stratified by *EGFR* fusion status were analyzed by the Wilcoxon test. The landscape of the co-occurring gene alteration events in a 539-gene panel was generated using ComplexHeatmap (R package). The circos plot showing the chromosome distribution of *EGFR* fusion was analyzed by RCircos (R package). The MutationMapper module (https://www.cbioportal.org/mutation_mapper) from cBioPortal was used to investigate the distribution of mutations at the protein domain. The Kaplan‒Meier curve analysis of OS was compared using the log-rank test. All reported P values were two-tailed, and *P* < 0.05 was considered statistically significant.

Results.

### Clinical characteristics of patients with *EGFR* fusion

**Fig. 1:**
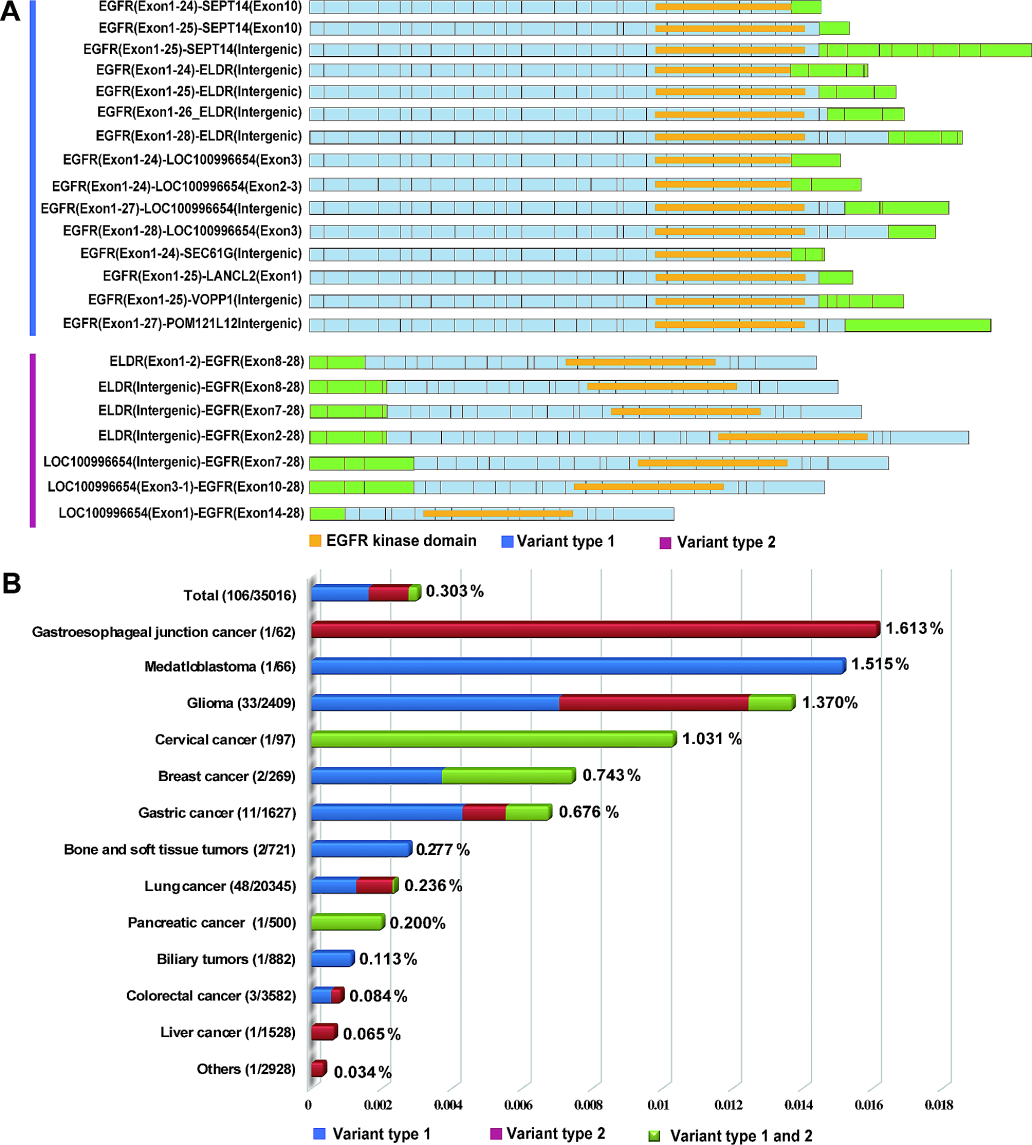
*EGFR* rearrangements in solid tumors. (**A**) Schematic of all *EGFR* fusions of two variant types. The dark blue in the upper part represents variant type 1, and the red represents variant type 2. The partners are colored green, with the EGFR kinase domain colored yellow and other *EGFR* exons colored blue by individual boxes. (**B**) The frequencies of *EGFR* fusions in diverse tumor types. Blue represents variant type1, red represents variant type1 and green represents both two variant types

In total, 0.303% (106/35,023) of patients in our cohort harbored *EGFR* fusion. *EGFR* fusion is classified into variant type 1 and type 2 according to the direction of fusion and the principle of activation. Variant type 1 is *EGFR-X*, which may result in the loss of the *EGFR* autophosphorylation site in the C-terminal tail of the receptor [20]. Variant type 2 is the *“X-EGFR”* and has a common fusion activation mechanism in which the partner gene leads to the activation and continued expression of the EGFR protein. All 106 *EGFR* fusions included the full EGFR kinase domain, which is encoded by exons 18–24. As seen in Fig. 1A, we present a schematic with an *EGFR* partner gene occurred ratio higher than 4%. The activation and treatment effectiveness with TKI have been reported in both *EGFR* fusion variant types [22, 23]. Patient characteristics are summarized in Table 1. Patients’ age ranged from 5 to 82 years, with a median age of 60.5 years. Sixty-three (59.43%) patients were male, and 43 (40.57%) were female. A total of 51.89% had clinical-stage III/IV disease versus 5.66% with stage I-II disease, and other stages were unavailable (NA). Among the top two cancers, adenocarcinoma (31, 64.58%) predominates in lung cancer, and glioblastoma (16, 48.48%) predominates in glioma. Variant type 1 accounted for 53.77%, variant type 2 accounted for 37.74%, and contained both two variant types accounted for 8.49%.

**Table 1:** Baseline characteristics of patients with *EGFR* fusion (*n* = 106)

Characteristics	*N* (%)
Age, years	60.5(5–82)
Median (range)	
Male gender	63(59.43%)
Cancer type	
Liver cancer	1(0.94%)
Colorectal cancer	3(2.83%)
Biliary tumors	1(0.94%)
Pancreatic cancer	1(0.94%)
Lung cancer	48(45.28%)
Bone and Soft Tissue Sarcoma	2(1.89%)
Gastric cancer	11(10.38%)
Breast cancer	2(1.89%)
Cervical cancer	1(0.94%)
Glioma	33(31.13%)
‘medulloblastoma’	1(0.94%)
Gastroesophageal junction cancer	1(0.94%)
others	1(0.94%)
Stage	
I	3(2.83%)
II	3(2.83%)
III	18(16.98%)
IV	37(34.91%)
NA	45(42.45%)
Pathology	
Lung cancer	48
Adenocarcinoma, ADC	31(64.58%)
squamous cell carcinoma, SQCC	2(4.17%)
NA	
Glioma	15(31.25%)
Astrocytoma	33
Glioblastoma	5(15.15%)
NA	16(48.48%)
	12(36.36%)
Fusion variant type	
Variant type 1	57(53.77%)
Variant type 2	40(37.74%)
Variant types 1 and 2	9(8.49%)

In total, *EGFR* fusions were detected in 0.303% (106/35,023) of the patients, with varying frequencies across diverse tumor types. Lung cancer exhibited a prevalence of 0.236% (48/20,345), glioma 1.370% (33/2,409), gastric cancer 0.676% (11/1,627), colorectal cancer 0.084% (3/3,582), breast cancer 0.743% (2/269), bone and soft tissue sarcoma 0.277% (2/721), and a single case of *EGFR* fusion was identified in gastroesophageal junction cancer, medulloblastoma, cervical cancer, pancreatic cancer, biliary tumors, liver cancer, and other types as shown in Fig. 1B.

### Identification of *EGFR* fusion partners in patients with different cancer types

**Fig. 2:**
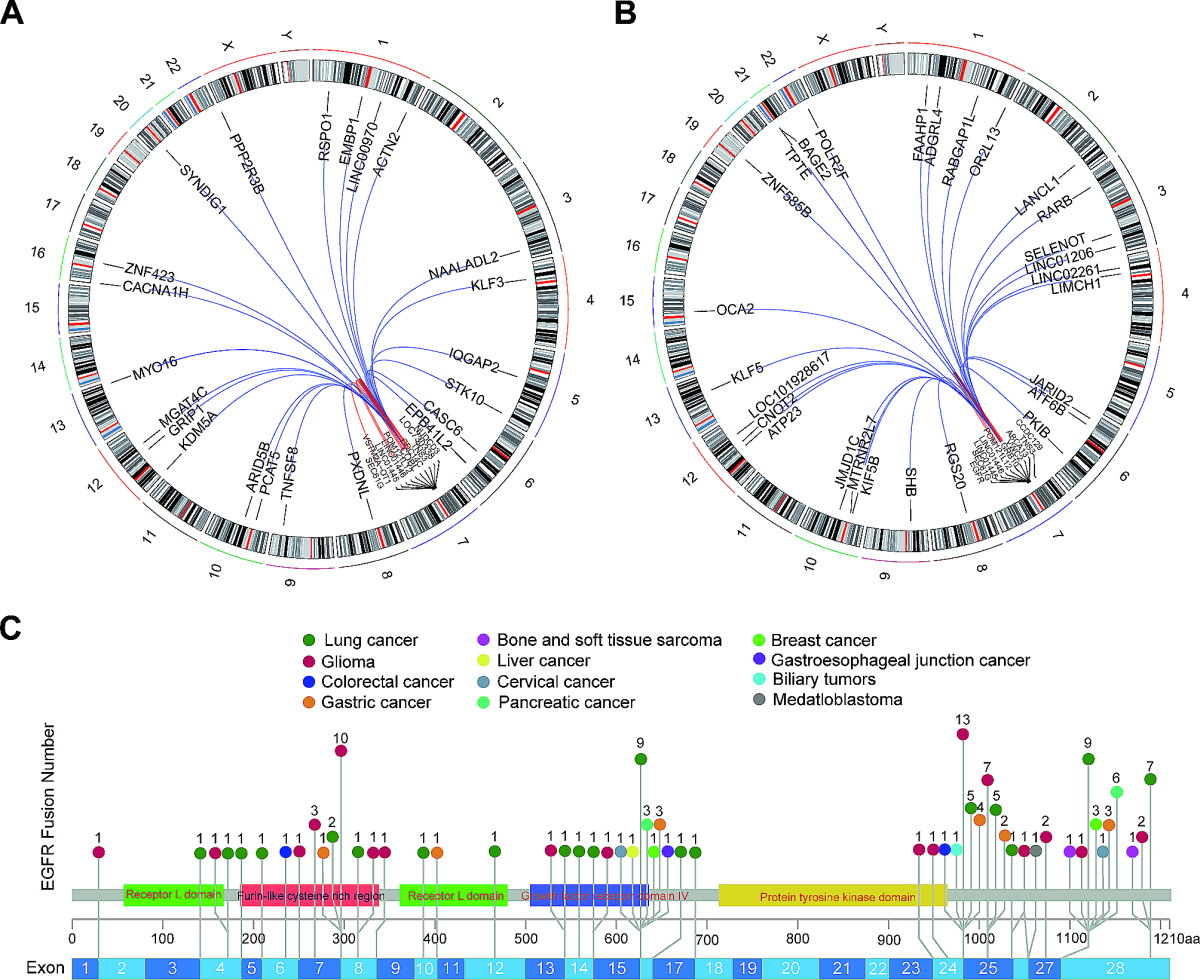
(**A**) Breakpoint positions of different *EGFR* fusion variant types. (**B**) Different colors represent different cancer types. (**C**) Numbers beyond circles represent the counts of functional *EGFR* fusions detected in different cancer type

The partner genes of the two fusion types are shown in Table 2. *SEPT14*, *ELDR*, and *LOC100996654* are the most frequent in variant type (1) *ELDR* and *LOC100996654* are the most common in variant type (2) The fusion partner genes are also different in various cancer types. The partner genes in different cancer types are shown in Supplementary Fig. 1A-B. The chromosome distribution of the fusion partner genes of the two types is shown in Fig. 2A-B and the partner genes are scattered across the chromosomes. Breakpoint positions of different *EGFR* fusion variant types are shown in Fig. 2C. The breakpoints of *EGFR* in patients with lung cancer were concentrated in exons 15, 16, 27, and 28. In patients with glioma, breakpoints of *EGFR* were concentrated in exons 7, 8, 24, and 25. The breakpoints of *EGFR* in other cancer types are scattered.

**Table 2:** Distribution of fusion partners identified in cancer patients with EGFR fusions

Variant type 1		Variant type 2	
Gene	Number	Gene	Number
SEPT14	10	ELDR	4
ELDR	6	LOC100996654	3
LOC100996654	5	HPVC1	2
LANCL2	4	LINC01446	2
POM121L12	4	POM121L12	2
FKBP9P1	3	RNR2	2
MYO16	3	ABCA13	1
SEC61G	3	ADGRL4	1
LINC01446	3	ASL	1
NUDCD3	2	ATF6B	1
VSTM2A-OT1	2	ATP23	1
ZNF713	2	BAGE2	1
ZNF733P	2	CCDC129	1
LINC01445	2	CNOT2	1
VOPP1	2	FAAHP1	1
ACTN2	1	FAM133DP	1
ARID5B	1	GRB10	1
CACNA1H	1	JARID2	1
CASC6	1	JMJD1C	1
CDC14C	1	KIF5B	1
COBL	1	KLF5	1
DNAJC2	1	LANCL1	1
EMBP1	1	LANCL2	1
EPB41L2	1	LIMCH1	1
GRIP1	1	LINC01206	1
IQGAP2	1	LINC01445	1
KDM5A	1	LINC02261	1
KLF3	1	LOC101928401	1
LINC00970	1	LOC101928617	1
LOC730338	1	LOC102723376	1
MGAT4C	1	MTRNR2L7	1
NAALADL2	1	OCA2	1
PCAT5	1	OR2L13	1
PPP2R3B	1	PKIB	1
PXDNL	1	POLR2F	1
RABGEF1	1	PRSS45	1
RSPO1	1	RABGAP1L	1
STK10	1	RARB	1
SYNDIG1	1	RGS20	1
TAC1	1	SEC61G	1
TNFSF8	1	SELENOT	1
UBE3C	1	SHB	1
ZNF423	1	TNS3	1
TNS3	1	TPTE	1
		TYW1B	1
		VOPP1	1
		VWC2	1
		ZNF585B	1

### Co-occurrence genetic mutants and TMB of patients harboring *EGFR* fusion

**Fig. 3:**
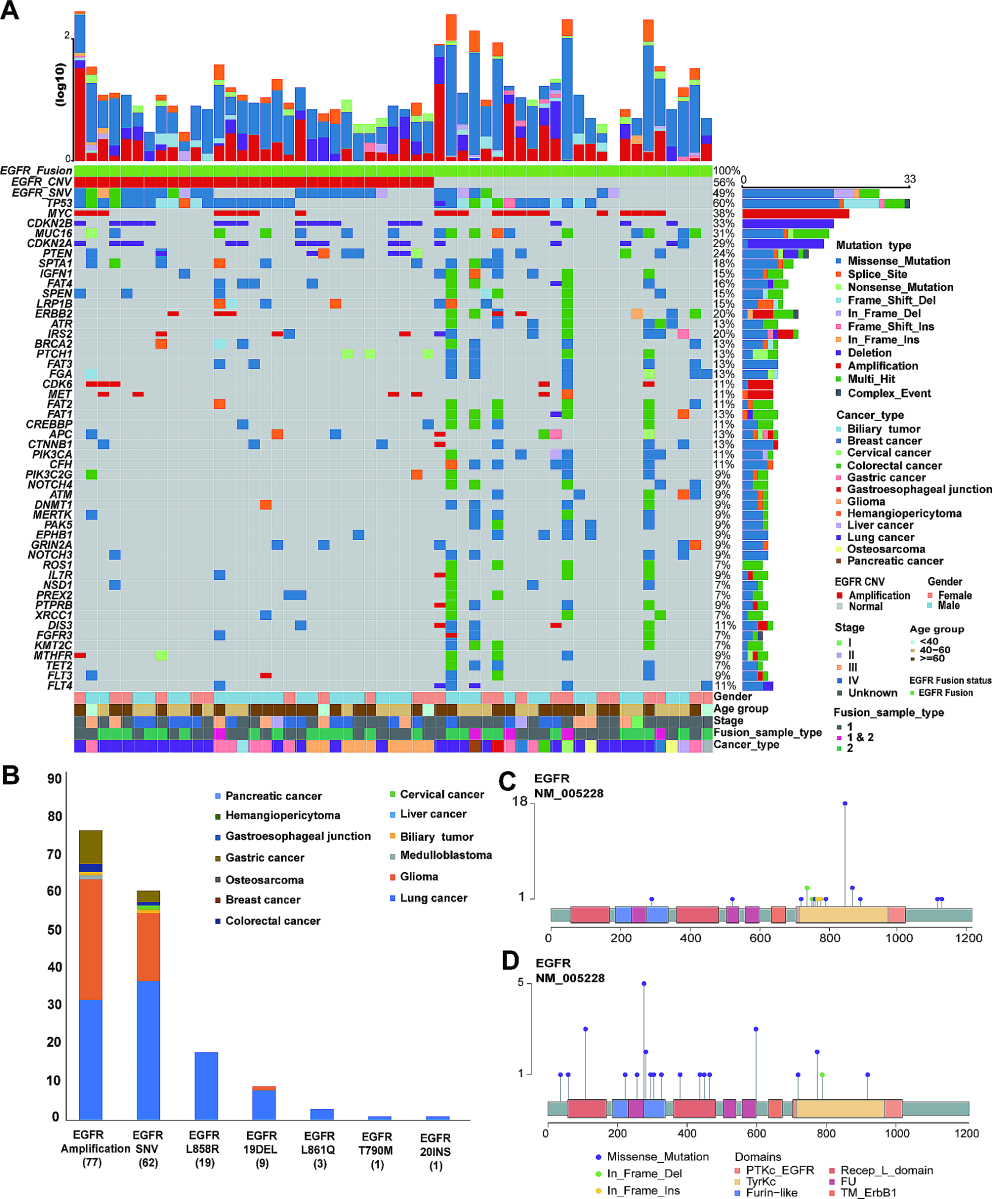
Heatmap of the co-mutation status of 55 patients whose tumors were subjected to NGS detection using a 539-gene panel (**A**). Gene mutation types in 106 *EGFR* fusion patients (**B**). The *EGFR* mutation sites are different in lung cancer (**C**) and glioma (**D**)

Among 106 patients with *EGFR* fusion, 55 patients’ tumors were examined by NGS using a 539-gene panel, and the co-mutation status of these patients is shown in Fig. 3A. Among the 55 patients with *EGFR* fusion, 31 (56%) patients had *EGFR* amplification, and 26 (47%) patients had *EGFR* single nucleotide variants (SNV, including missense mutation, in-frame deletion, in-frame insertion and multi-hit) mutations. Notably, there were 19 (35%) patients with sensitive mutations. The top 5 genes co-mutated with *EGFR* fusion were *TP53* (mutation frequency, MF: 65%), *BRCA2* (MF: 43%), *ALK* (MF: 41%), *MUC16* (MF: 39%), and *MYC* (MF: 39%).

The median TMB among the 55 patients with *EGFR* fusion was 4.26 (0.71-139.01) Muts/Mb in solid tumors. The median TMB was 3.97 (0.71-139.01) in the 26 patients with lung cancer, 9.22 (1.42–63.83) in the nine patients with gastric cancer and 2.49 (0.71–4.26) in the eight patients with glioma. There was no significant difference in TMB between *EGFR* fusion and non-*EGFR* fusion groups, whether among pan-cancer (*p* = 0.054), lung cancer (*p* = 0.28), glioma (*p* = 0.27) or gastric cancer patients (*p* = 0.11, Supplementary Fig. 2).

Among 106 patients with *EGFR* fusion, there were 77 patients with *EGFR* CNV and 62 patients with *EGFR* SNV (including L858R, 19 exon deletion, L861Q, T790M, 20 exon insertion and so on; Fig. 3B). *EGFR* L858R, L861Q, T790M, and 20 exon insertion are mutation types that have only been detected in lung cancer patients in our cohort, while other types of cancer have not been detected. Furthermore, according to Fig. 3C **and D**, the *EGFR* mutation sites in lung cancer and glioma are different. In lung cancer, *EGFR* mutations mostly occurred in functional kinase domain, mainly tyrosine kinase catalytic (TyrKc). But in glioma, the most common *EGFR* mutation is *EGFRvIII* mutation, which may be caused by deletion of exons 2–7. These results are basically consistent with the results reported in other previous studies [14–16, 19].

### The prognostic impact of *EGFR* fusions

We collected 9,563 tumor variant data in 30 cancer types from TCGA to calculate the proportion of *EGFR* fusions, and 27 (0.28%) samples were identified harboring *EGFR* fusions in six cancer types, including 22 patients with variant type 1 and 5 patients with variant type 2. The proportion of glioma was the highest (16/1094, 1.46%), followed by bladder urothelial carcinoma (3/410, 0.73%) and head and neck squamous cell carcinoma (3/522, 0.57%) (Table 3). Notably, there was no co-mutant *EGFR*-sensitive mutation, but 26 *EGFR* CNV co-mutations were found among the 27 patients with *EGFR* fusion.

**Table 3:** The frequency of *EGFR* fusion in different cancer types in TCGA

Cancer type	Patients (*N*)	EGFR fusion (*N*)	Percentage (%)
Glioma	1094	16	1.46
Bladder Urothelial Carcinoma	410	3	0.73
Head and Neck squamous cell carcinoma	522	3	0.57
Stomach adenocarcinoma	435	2	0.46
Lung cancer	986	2	0.20
Liver hepatocellular carcinoma	371	1	0.27
Others	5745	0	0

**Fig. 4:**
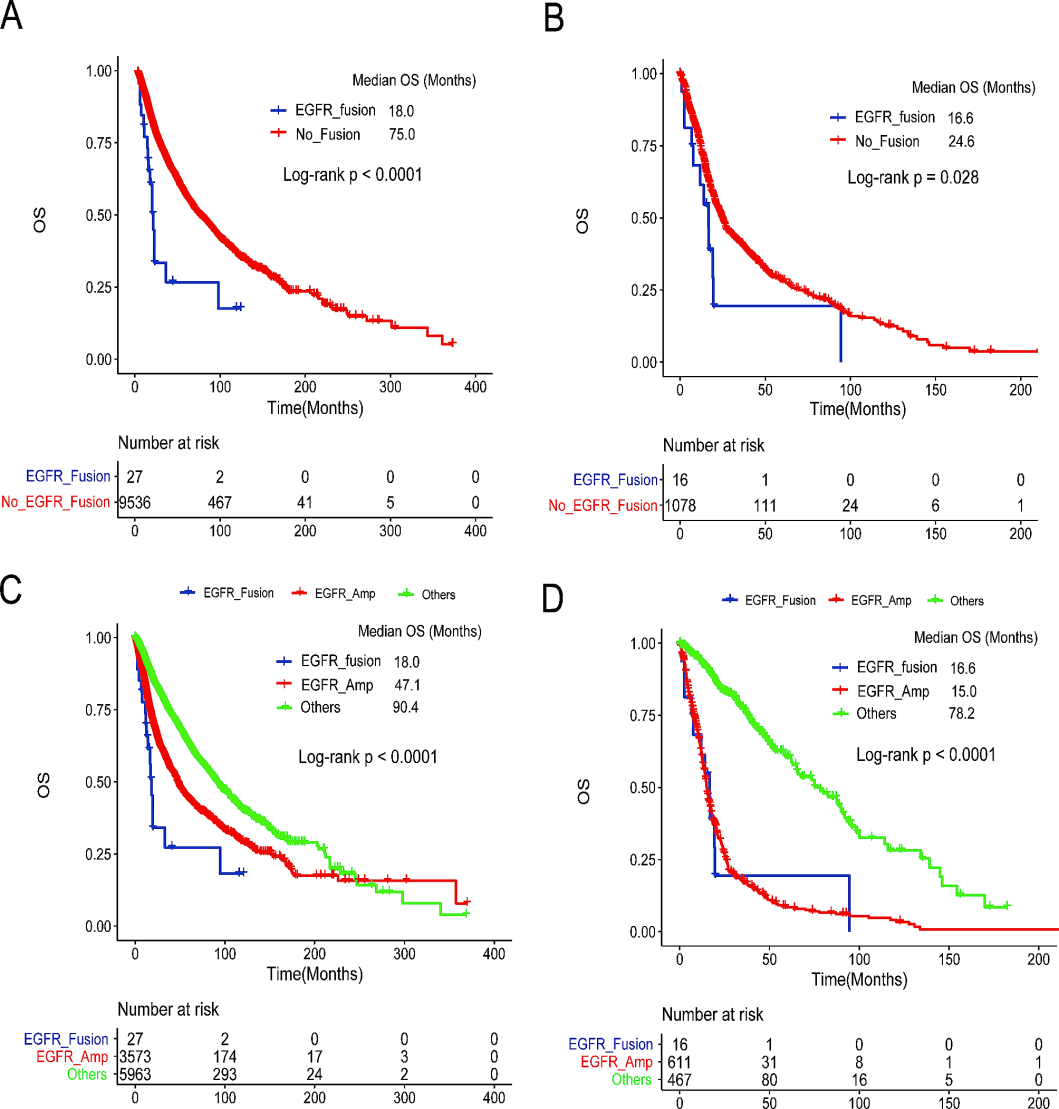
Kaplan‒Meier survival curves of patients with pan-cancer TCGA. (**A**) Kaplan‒Meier survival curves of patients with pan-cancer in the *EGFR* fusion group and non-EGFR fusion group in TCGA in pan-cancer. (**B**) Kaplan‒Meier survival curves of patients with pan-cancer in the *EGFR* fusion group and non-EGFR fusion group in TCGA in glioma. (**C**) The Kaplan‒Meier survival curves of patients with pan-cancer in the *EGFR* fusion with CNV co-mutation group and only *EGFR* CNV group in TCGA. (**D**) The Kaplan‒Meier survival curves of patients with pan-cancer in the *EGFR* fusion with CNV co-mutation group and only *EGFR* CNV group in TCGA

We further explored the relationship between *EGFR* fusion and overall survival (OS) in the TCGA cohort. Patients with *EGFR* fusion had a significantly shorter OS than those without *EGFR* fusion in pan-cancer (*p* < 0.0001, Fig. 4A) and in glioma (*p* = 0.028, Fig. 4B), suggesting that *EGFR* fusion might be a high-risk factor for poor prognosis. Notably, there were 26 *EGFR* amplification co-mutations among the 27 *EGFR* fusion samples. Therefore, we specifically detected the relationship between *EGFR* fusion, only the *EGFR* amplification group and others (without *EGFR* fusion and *EGFR* amplification). The results showed that in pan-cancer, the *EGFR* fusion group had the worst OS, and the *EGFR* CNV group had a comparably worse OS than others group (*p* < 0.0001, Fig. 4C). In glioma, the *EGFR* fusion and the *EGFR* amplification groups had a comparably worse OS than others group (*p* < 0.0001, Fig. 4D).

## Case presentation

**Fig. 5:**
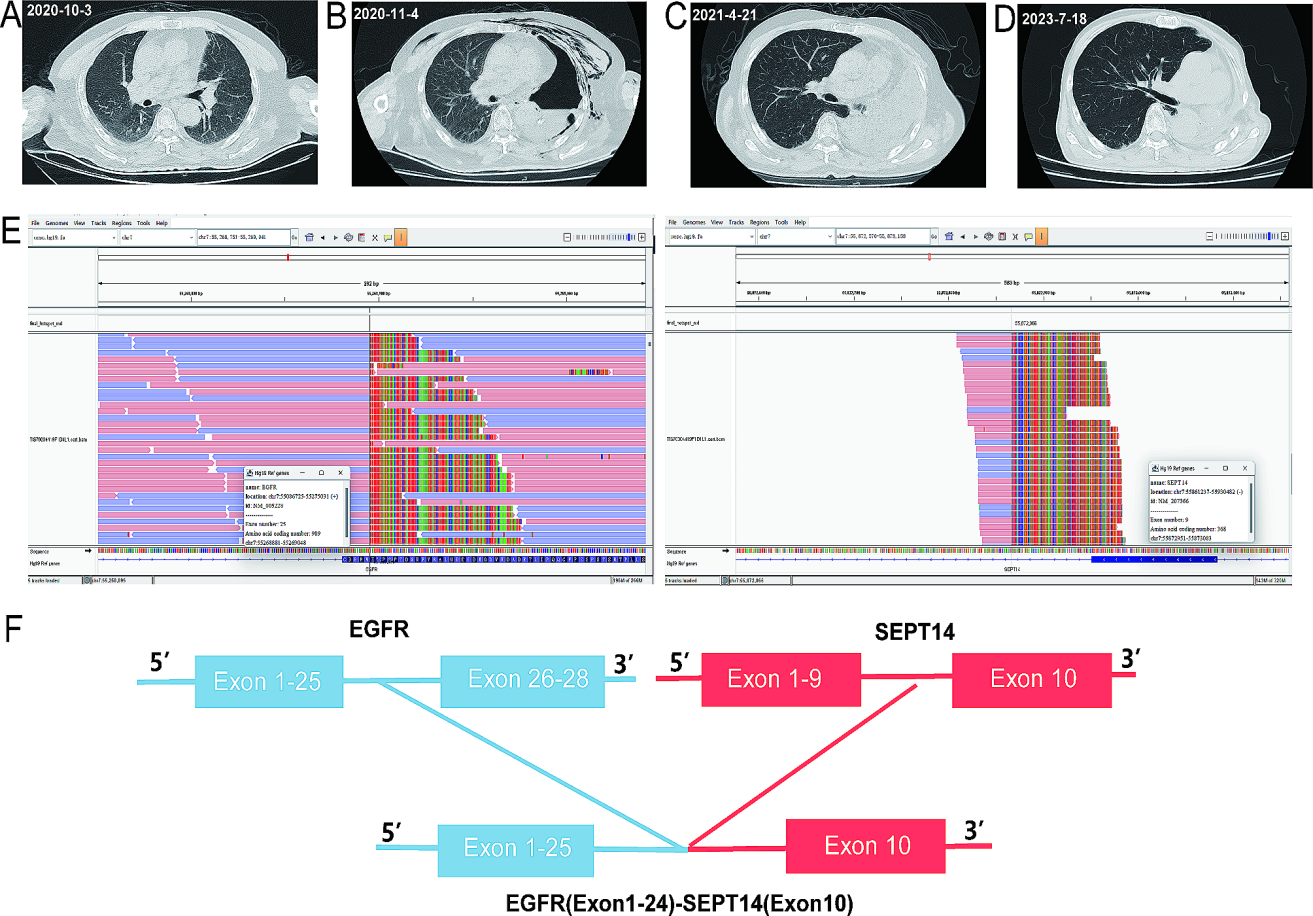
Chest computed tomography revealed the different stages of the patient’s lung tumor and Next-generation sequencing findings of *EGFR-SEPT14* fusion. (**A**) Computed tomography diagnosis of lung tissue. (**B**) CT scan after the surgery. (**C**) CT scan after adjuvant chemotherapy. (**D**) CT scan after almonertinib treatment for 26 months. (**E**) Sequencing reads of *EGFR* are shown by the Integrative Genomics Viewer. (**F**) Illustration of the *EGFR-SEPT14* fusion

A 71-year-old male patient was admitted to the hospital for 15 days with an irritant cough and two days with bloody sputum. A space-occupying lesion in the left upper lobe was discovered using computed tomography (CT) (Fig. 5A). Following the appropriate preoperative examination, a left total pneumonectomy was performed on November 2, 2020 (Fig. 5B). Percutaneous lung biopsy yielded a diagnosis of lung adenocarcinoma. Finally, the disease was diagnosed as stage IIIA (T1bN2M0). The patient then received postoperative adjuvant chemotherapy for six months, CT results were shown in Fig. 5C. Subsequently, FFPE samples were analyzed by NGS, and a novel *EGFR-SEPT14* (11.36% abundance) fusion was detected (Fig. 5E-F). Several other mutations, such as *TP53*,* CCNE1* and *HRAS* were also observed (Supplementary Table 1). The patient then received almonertinib mesilate tablets (500 mg daily) and remained relapse-free until July 8, 2023 (Fig. 5D).

## Discussion

Our study is the first extensive sample study analysis of *EGFR* fusion in solid tumors. We retrospectively analyzed *EGFR* fusion among 35,023 patients with solid tumors who underwent NGS detection and found that the overall incidence of *EGFR* fusion in solid tumors was 0.303%. A total of 106 patients were found harboring *EGFR* fusion, of which 57 patients harbored variant type 1, 40 harbored variant type 2, and 9 harbored both types 1 and 2. The incidence of *EGFR* fusion in various cancer types was quite different, ranging from 0.034 to 1.613%. The incidences of glioma, gastric cancer, and lung cancer with a large number of participants were 1.37% (33/2409), 0.676% (11/1627), and 0.236% (48/20,345), respectively. This is the first time that the frequency of the *EGFR* fusion has been disclosed in detail.

*EGFR* fusion represents a novel oncogenic driver across different cancer types. There could be two distinct activation mechanisms of *EGFR* fusion. The variant type 1 is *EGFR-X*, and the mechanism has been reported to be that the loss of the C-terminal tail autophosphorylation site leads to the loss of the Cbl binding site, which makes the EGFR fusion protein more stable and finally activates tumorigenic signaling and forms an oncogenic phenotype [20, 32]. This activation mechanism of variant type 1 has been reported, and several cases are successful against EGFR TKI, including in lung cancer and colorectal cancer [20, 23, 33, 34]. Variant type 2 is *X-EGFR*, and its activation mechanism is similar to that of *ALK* fusion, in which *EGFR* retains the complete kinase domain and the partner gene may contain promoters or CC domains. Preclinical carcinogenic effects of this fusion have also been reported [24], as well as cases of TKI sensitivity [22, 35]. Gene rearrangement plays an essential role in the occurrence and development of tumors, and rearrangement can be treated by TKI targeting with generally satisfactory effects. Both variant types 1 and 2 can activate EGFR and operate as effective TKI targets, making them novel therapeutic targets for patients with solid tumors.

The partner gene was further investigated, and the distribution of *EGFR* partner genes was found to be relatively scattered across the chromosome. Variant types 1 and 2 are evenly distributed on chromosomes. Unlike the case report, there is no *RAD51* gene, which is more reported in other literature. In addition to some reported *EGFR* fusions, including *EGFR-ZNF713*, *EGFR-TNS3*, and *EGFR-SEPT14*, various rare and novel *EGFR* fusion partners were identified in this study. In addition, multiple *EGFR* fusions were identified in one individual patient. The distribution of *EGFR* partner genes in each cancer species is also scattered, which may introduce challenges for the widespread detection of partners. NGS can accurately identify fusion partners and breakpoints, which may become the most widely used procedure in clinical practice.

Previous reports of *EGFR* fusions were based mainly on the occurrence and cases of lung cancer. Our study is the first large-scale retrospective study of *EGFR* fusion occurrence in pan-solid tumors. The frequencies of *EGFR* fusions in pan cancer and some cancers are similar in Chinese and Western populations (TCGA), including glioma (1.37% vs. 1.46%) and lung cancer (0.236% vs. 0.20%). In addition, we analyzed the co-mutations of *EGFR* fusion, including the combination of *EGFR*-sensitive mutations and *EGFR* CNV, of which 30 (56%) patients had *EGFR* CNV and 19 (35%) patients had *EGFR-*sensitive mutations in our cohort. However, the combination of *EGFR*-sensitive mutations is different from that of TCGA, which may be related to ethnic differences, and Asians have a higher frequency of *EGFR*-sensitive mutations. Additionally, the TMB of *EGFR* fusion patients was further analyzed, which indicated that the TMB of *EGFR* fusion was higher than that of non-*EGFR* fusion but with no statistical significance, no matter in pan cancer or in the lung, gastric cancer, and glioma.

Patients with *EGFR* fusion had a significantly shorter OS than patients without *EGFR* fusion in TCGA, which suggests that *EGFR* fusion is probably a high-risk factor for poor prognosis. Emerging evidence supports that *EGFR* amplification predicts worse outcomes in patients with lung cancer [36]. Almost all patients with *EGFR* fusion had *EGFR* amplification (96.30%, 26/27) in TCGA. We explored the relationship between *EGFR* fusion and only *EGFR* CNV in the TCGA cohort. Patients in the *EGFR* fusion group had a significantly shorter OS than those in the group only harboring *EGFR* amplification, which means that patients with *EGFR* fusion have a worse prognosis.

A few of cases have been reported in which patients with *EGFR* fusions responded to corresponding EGFR-TKIs, including erlotinib, icotinib, and afatinib [21–24]. Guoqing Zhang et al. described a patient with NSCLC with *EGFR* fusion and *EGFR* amplification who achieved a significant antitumor response from treatment with gefitinib combined with cetuximab [37]. Our case described success in treating with EGFR-TKIs, which provides a reference for future EGFR-TKI clinical trials with *EGFR* fusions.

However, some limitations are worth noting. There was no RNA-based NGS or protein level validation to confirm the functional activation of *EGFR* fusion. Some cancer types had limited numbers of patients, and clinical follow-up data were unavailable from our cohort. The distribution of partner genes is not concentrated, and future detection of *EGFR* fusion needs to be carried out by NGS.

In summary, this is the first retrospective analysis of *EGFR* fusions in a large-scale solid tumor population. Our study may provide a reference for designing future EGFR TKI clinical trials with *EGFR* fusions.

### Electronic supplementary material

Below is the link to the electronic supplementary material.

Supplementary Material 1

Supplementary Material 2

Supplementary Material 3

## Data Availability

No datasets were generated or analysed during the current study.
