# Supplementary figures and images for: Distribution of EGFR fusions in 35,023 Chinese patients with solid tumors-the frequency, fusion partners and clinical outcome
Source: World J Surg Oncol. 2024 Jul 25;22:194. doi: 10.1186/s12957-024-03463-w (PMC11271172; doi:10.1186/s12957-024-03463-w)

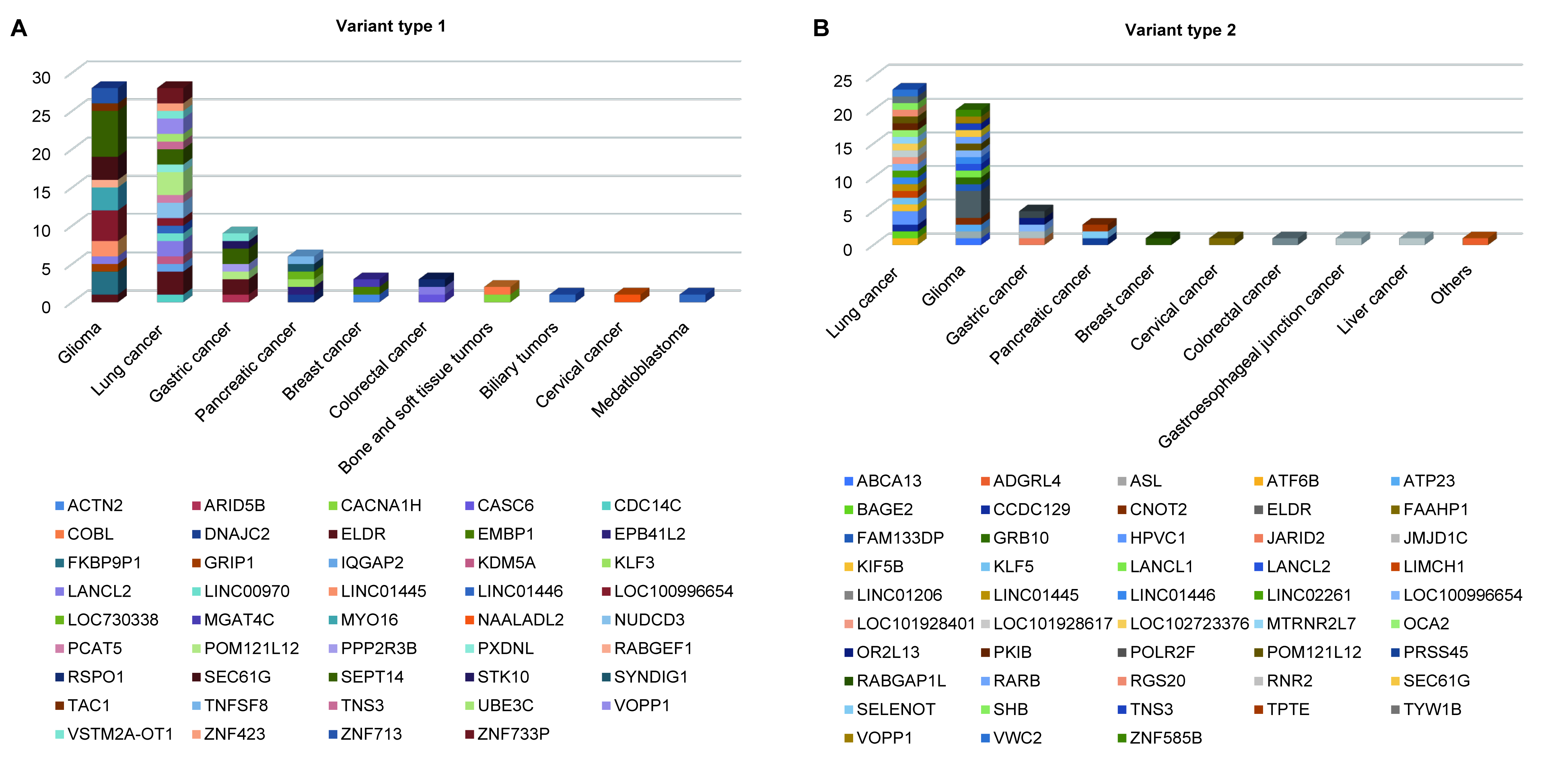

Supplement: Supplementary file 1 — Supplementary Material 1 [file 12957_2024_3463_MOESM1_ESM.tif]

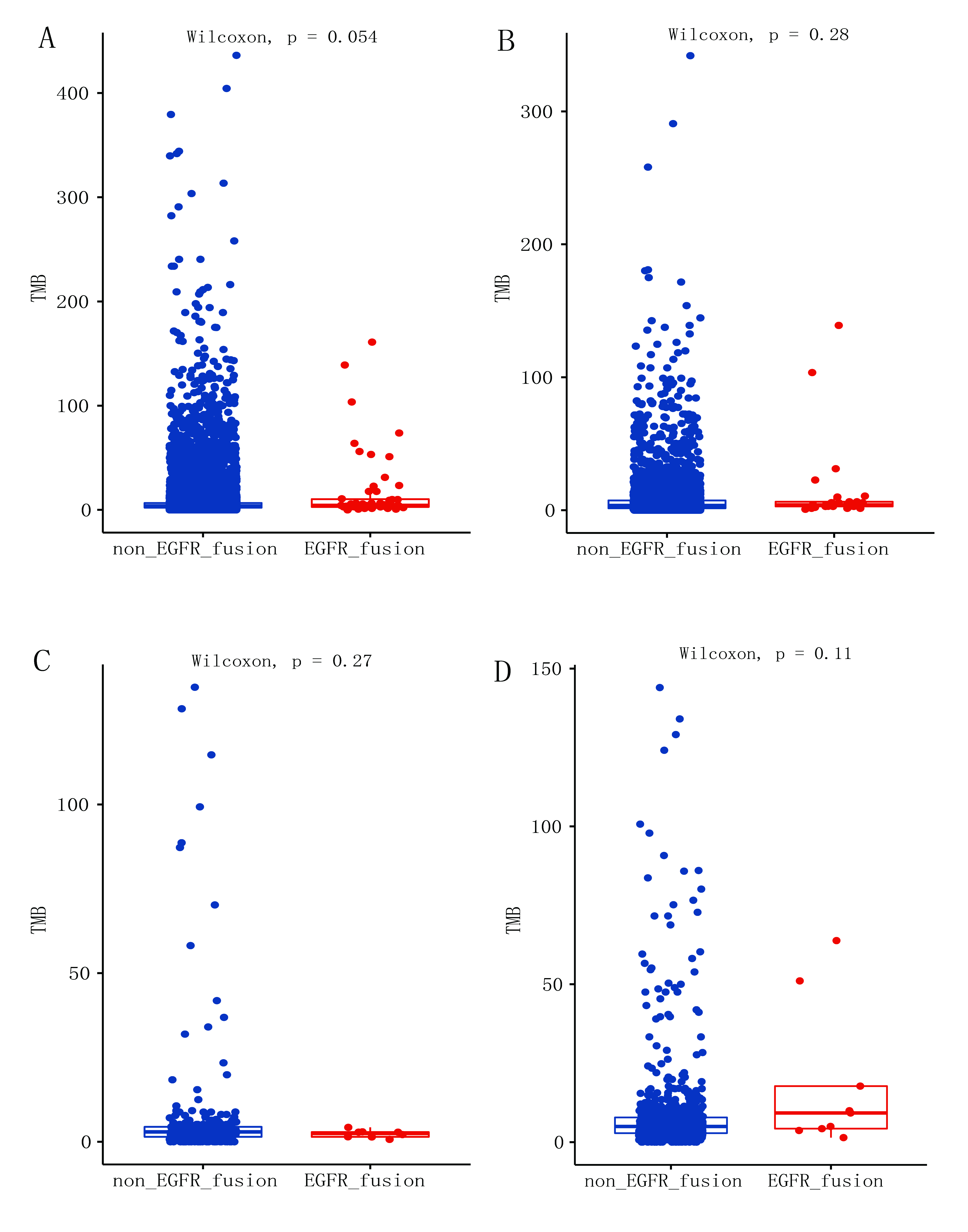

Supplement: Supplementary file 2 — Supplementary Material 2 [file 12957_2024_3463_MOESM2_ESM.tif]
