# Supplementary material for: Distribution of EGFR fusions in 35,023 Chinese patients with solid tumors-the frequency, fusion partners and clinical outcome
Source: World J Surg Oncol. 2024 Jul 25;22:194. doi: 10.1186/s12957-024-03463-w (PMC11271172; doi:10.1186/s12957-024-03463-w)
Supplement: Supplementary file 3 — Supplementary Material 3 [file 12957_2024_3463_MOESM3_ESM.docx]

Supplementary Table 1 Next-generation sequencing analysis was performed usingformalin-fixed and paraffin-embedded specimen (FFPE) sample.

| **Gene name** | **Mutation** | **Abundance/**  **copy number** |
| --- | --- | --- |
| *TP53* | exon7 c.716delA p.N239Tfs*8 | 23.08% |
| *EGFR-SEPT14* | Fusion | 11.36% |
| *CCNE1* | Amplification | 3.95 |
| *HRAS* | Amplification | 3.45 |
| *RBM10* | exon16 c.1784A>G p.Q595R | 24.81% |
| *HGF* | exon13 c.1514T>G p.I505R | 19.8% |
| *SMAD4* | exon9 c.1082G>A p.R361H | 9.17% |
| *PBRM1* | exon27 c.4230delG p.P1411Lfs*21 | 8.1% |
| *DDR1* | exon3 c.332G>C p.G111A | 7.4% |
| *ERBB4* | exon1 c.35G>C p.S12T | 7.06% |
| *HGF* | exon16 c.1862G>T p.G621V | 5.35% |
| *ATM* | exon35 c.5269A>G p.T1757A | 4.43% |
| *TERT* | Amplification | 4.33 |
